# Distinct patterns of endosulfatase gene expression during *Xenopus laevis* limb development and regeneration

**DOI:** 10.1002/reg2.27

**Published:** 2015-03-13

**Authors:** Yi‐Hsuan Wang, Caroline Beck

**Affiliations:** ^1^ Department of ZoologyUniversity of OtagoPO Box 56DunedinNew Zealand

**Keywords:** development, endosulfatase, expression, regeneration, *Xenopus*

## Abstract

The heparan sulfate 6‐*O*‐endosulfatases sulf1 and sulf2 regulate multiple cellular processes and organ development. Sulfs modulate a range of heparan‐sulfate‐dependent extracellular pathways, including the fibroblast growth factor, bone morphogenetic protein, and wingless/wnt signaling pathways. Known patterns of sulf transcript expression together with functional experiments have implicated the sulfs in chondrogenesis and muscle regeneration in mammals. Here, we describe the expression patterns of *Xenopus laevis sulf1* and *sulf2* in developing forelimbs and hindlimbs and demonstrate novel expression of the *sulf* transcripts in the regenerating hindlimbs, with prominent *sulf2* expression in the proliferating blastema and transient expression of *sulf1* in the redeveloping apical epidermal ridge. These findings further suggest involvement of the sulfs in successful limb regeneration in amphibians.

## Introduction

The process of wound healing as well as epimorphic regeneration of a lost structure or organ in an amphibian model requires extensive proliferation of cells and a certain degree of cellular plasticity for its success. In the case of wound healing, the layer of wound epithelium that forms over the surface of trauma originates from the underlying skin epidermis. In regeneration competent limb stages, the wound epithelium then thickens to form a multilayered apical epithelial cap, which is the morphologic and functional equivalent of the apical ectodermal ridge (AER) in amniote and anuran limb buds (Barker and Beck 2009; Beck 2012). This is followed by the formation of the blastema from the mesenchymal cells below the epithelium layer, which then proliferates and eventually reforms the lost limb structure (Barker & Beck 2009). Numerous growth factors and a host of other developmental genes are associated with the regulation of cell proliferation, migration, and differentiation in both the developing and regenerating limbs (Towers & Tickle 2008; Zeller et al. 2009).

Past studies have shown that heparan sulfate proteoglycans (HSPGs) play a role in the regulation of a number of signaling pathways that are critical for cell differentiation and normal animal development (Kirn‐Safran et al. 2009; Yan & Lin 2009). HSPGs normally consist of a number of core proteins that are covalently linked to at least one heparan sulfate (HS) chain, which is subject to post synthetic modification along its chain of disaccharide units via sulfation by various enzymes depending on the cellular context (Gorsi & Stringer 2007; Kirn‐Safran et al. 2009; Rosen & Lemjabbar‐Alaoui 2010; Turnbull 2010). The variety of structural and signaling functions of HSPGs can be attributed to their ability to bind to a large number of growth factors, morphogens, matrix proteins, and cell adhesion molecules. Ligand binding by HSPGs is influenced by the structure of the HS chains, especially the density and pattern of sulfation modifications (Kirn‐Safran et al. 2009; Rosen & Lemjabbar‐Alaoui 2010). Post synthetic modification of HS chains by two extracellular endosulfatases (hereafter referred to as sulfatases), in particular, have been shown to produce specific physiological effects including general cell development, angiogenesis, and other biological processes (Gorsi & Stringer 2007 Lamanna et al. 2007).

The HS 6‐*O*‐endosulfatases *Sulf1* and *Sulf2* play important roles in cellular processes and organ development by modulating the activity of fibroblast growth factors (Fgf), bone morphogenetic proteins (Bmp), wingless (Wnt) and other HS‐dependent extracellular signaling pathways (Dhoot et al. 2001; Viviano et al. 2004; Wang et al. 2004; Morimoto‐Tomita et al. 2005; Otsuki et al. 2010; Ramsbottom et al. 2014). They were originally identified in muscle and neural progenitors of quail by Dhoot et al. (2001), are capable of remodeling the internal 6‐*O*‐sulfation levels within intact HS extracellularly, and are the only HS‐editing enzymes that modify the sulfated HS sequences after HS biosynthesis (Ai et al. 2003, 2006; Turnbull 2010; Tran & Vleminckx 2013). The *Drosophila sulf1* modulates the Wnt morphogen gradients in developing fly larvae as well as hedgehog (Hh) signaling during wing development by lowering the concentration of the related morphogens in the wing discs (Kleinschmit et al. 2010; Wojcinski et al. 2011). In quail, the expression of sulf1a is required for the activation of *MyoD*, a Wnt‐induced regulator of muscle specification during embryonic development (Dhoot et al. 2001). It also promotes the formation of low affinity HS−wnt complexes that can functionally interact with the frizzled receptors to initiate Wnt signal transduction via 6‐*O*‐desulfation from the HS chains on the cell surface (Ai et al. 2003, 2006). *Xenopus tropicalis Sulf1* and *Sulf2* are capable of enhancing the axis‐inducing activity of Wnt‐11, while restricting Bmp and Fgf signaling in cell movement and differentiation during embryogenesis and organogenesis (Isaacs et al. 1992; Hongo et al. 1999; Freeman et al. 2008).

Not surprisingly, since HSPGs interact with signaling molecules and morphogens, such as Hh, Fgf, and Bmp, that regulate cell differentiation and development in general, they are also implicated in both limb development and regeneration. Sulfs play a role in multiple stages of limb skeletal and muscle development, as indicated by the expression of the genes in the limb buds of mice and quail (Zhao et al. 2006; Ratzka et al. 2008; Otsuki et al. 2010). The highest level of quail *Sulf1a* expression in the limb buds is observed in condensing mesenchyme during the early differentiation stage of chondrogenesis, with a highly dynamic expression observed in the perichondral and joint‐forming regions as the autopod develops (Zhao et al. 2006). In mice, the expression of sulfs simultaneously enhances Bmp but inhibits Fgf signaling in chondrocytes, and thereby maintains cartilage homeostasis, as spontaneous cartilage degeneration and surgically induced osteoarthritis were significantly more severe in *sulf1^−/−^* and *sulf2^−/−^* mice compared with wildtype mice (Otsuki et al. 2010). Fine‐tuned expression of *sulfs* throughout limb development is essential for mesenchymal condensation and early differentiation of chondrocytes and other cartilaginous elements, as well as the subsequent joint formation and digital development in the autopod (Zhao et al. 2006; Ratzka et al. 2008; Otsuki et al. 2010). Compound mice mutants of *sulf1* and *sulf2* showed subtle but distinct skeletal malformation, including reduced bone length and fusions of sternebrae and tail bone (Ratzka et al. 2008). The growth‐factor‐dependent expression and functioning of mouse Sulfs, particularly mouse Sulf1, play a part in the regulation of growth factor signaling for satellite cell (SC) differentiation and skeletal muscle regeneration by promoting canonical Wnt signaling and enhancing myoblast fusion in the injured tissue (Langsdorf et al. 2007). Similarly, mouse Sulfs have also been shown to promote migration of corneal epithelial cells during wound repair by modulating the Wnt/β‐catenin signaling in mice (Maltseva et al. 2013).

This study provides the first in‐depth description of *sulf1* and *sulf2* expression in *Xenopus laevis* limbs during normal limb development and hindlimb regeneration. The general patterns of *Xenopus sulf1* and *sulf2* expression in developing limbs demonstrate similarity with the known patterns of the mammalian sulf expression in neonatal mice, with subtle differences in the temporal and spatial patterns (Ratzka et al. 2008). The apparent differences in the expression patterns of the two sulfatases in the respective developing and regenerating hindlimbs also suggest alternative functions of the genes during regeneration.

## Results

The *X. laevis sulf1* and *sulf2* probes were tested on stage 30−40 *X. laevis* embryos prior to experiment with limb samples, and the results correspond to the known pattern of expression of *Xenopus Sulf1* and *Sulf2* in *X. tropicalis* at similar stages (Fig. S1) (Winterbottom & Pownall 2009).

### Transient expression of *sulf1* in developing limb joints

**Figure 1:**
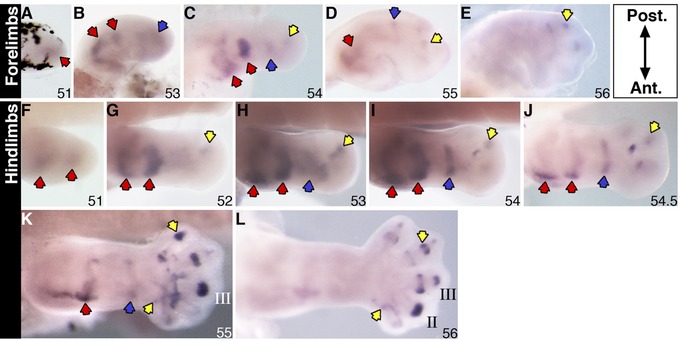
*Sulf1* expression in *X. laevis* stage 51−56 forelimbs (A−E) and stage 51−56 hindlimbs (F−L) examined by a whole mount in situ hybridization. Specific staining is dark purple. Red arrows in the more proximal part indicate gene expression at the putative shoulder/hip and elbow/knee joints; blue arrows indicate expression at the future wrist/ankle joints; yellow arrows indicate transcript expression in the autopod region. Roman numerals indicate digit number. All limbs are oriented proximal to the left and posterior uppermost, with the tadpoles lying on their right side.

Expression of *sulf1* transcripts was faintly visible in the distal mesenchyme of the stage 51 forelimb bud (Fig. 1A) and in the prospective hip and knee joints in the hindlimb bud (Fig. 1F; red arrows). Expression in the hip and knee joints in the hindlimbs, and shoulder and elbow joints in the forelimbs, became more distinct by stage 52−53 (Fig. 1B, G−H; red arrows), and staining of the joints in the autopod region became visible (Fig. 1B, G−H; blue and yellow arrows). By stage 54, the transcript expression in the wrist/ankle joints (blue arrows) and phalangeal joints (yellow arrows) in the forelimbs and hindlimbs was clearer in the early autopod (Fig. 1C, I−J). The overall level of expression across the forelimb bud appeared to regress at stage 55, while the staining for the gene remained faintly visible in the shoulder, wrist and autopod region (Fig. 1D). By stage 56, expression of *sulf1* was limited to the joint regions of digits II and III on the posterior side of the forelimb (Fig. 1E). In the late stage 54 hindlimbs, the more proximal expression of *sulf1* in the hip and knee joints regressed significantly across the posterior side, leaving a strong expression in the anterior side (Fig. 1J). The ankle expression also began to regress at stage 55 (blue arrow), whereas digital expression of *sulf1* appeared to be stronger and more distinct in the interphalangeal joints as well as in the digit‐to‐metatarsal joints in the autopod (Fig. 1K; yellow arrows). By stage 56, transcript expression of the gene was only visible in the autopod, with prominent expression in the digital joints (Fig. 1L). *Sulf1* expression in the stylopod and zeugopod regressed to the anterior after stage 54 (Fig. 1F−J), whereas the transcript level of expression in the digital joints appeared to become higher from there on (Fig. 1J−L). The expression in the autopod appeared to follow the order of digit formation as the *sulf1* transcripts were observed first in the putative digit IV position (Fig. 1H), then digits II and III, as well as progressing in a proximal to distal direction along the interphalangeal and phalangeal joints (Fig. 1I−L).

### *Sulf2* is expressed in developing autopod

**Figure 2:**
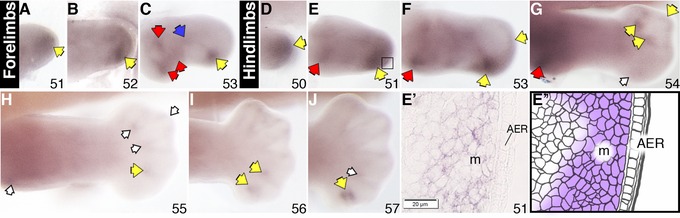
*Sulf2* expression in *X. laevis* stage 51−53 forelimbs (A−C) and stage 50−57 hindlimbs (D−J) examined by a whole mount in situ hybridization. Specific staining is dark purple. Red arrows in the more proximal part indicate gene expression at the putative shoulder/hip and elbow/knee joints; blue arrows indicate expression at the future wrist/ankle joints; yellow arrows indicate transcript expression in the autopod region; empty arrows indicate reduced level of expression. Roman numerals indicate digit number. The boxed region in E indicates area of histological section shown in E′, accompanied by the corresponding schematic (E″) showing area of gene expression in the mesenchyme. All limbs are oriented proximal to the left and posterior uppermost, with the tadpoles lying on their right side. AER, apical epidermal ridge; m, mesenchyme.

Transcript expression of *sulf2* was observed in the distal mesenchyme of stage 50 hindlimb buds and stage 51 forelimbs respectively (Fig. 2D and A). The expression of the gene in stage 51−53 hindlimb buds appeared strongest in the anterior distal mesenchyme (Fig. 2E and E′). The expression of the gene in stage 52−53 forelimbs also demonstrated an anterior bias in the distal mesenchyme (Fig. 2B, C). Additional anterior expression in the proximal and middle mesenchyme domain in hindlimbs was first seen at stage 51 and persisted to stage 53 (Fig. 2E, F). The level of overall transcript expression in the forelimb decreased from stage 54 onwards (data not shown). In stage 54 hindlimbs, the anterior expression in the distal and proximal part of the limb also regressed significantly, while staining in the interdigital space and distal mesenchyme in the posterior autopod was detected (Fig. 2G). The anterior−proximal expression in the hindlimb was barely visible by stage 55, whereas the interdigital expression of the gene became more obvious in the more differentiating autopod (Fig. 2H). By stage 56, transcript expression was only visible in the interdigital space, with a relatively higher level of staining in the anterior webbing (Fig. 2I−J).

### Re‐expression of *sulf1* and *sulf2* in regenerating hindlimbs

**Figure 3:**
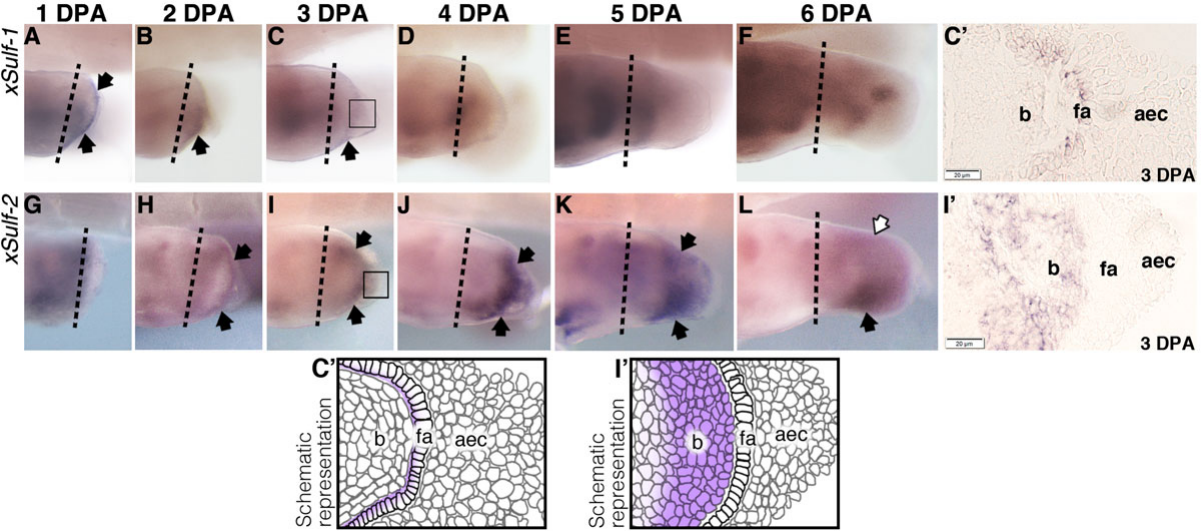
*X. laevis sulf1* (A−F) and *sulf2* (G−L) expression in regenerating hindlimbs originally operated at developmental stage 52 examined by a whole mount in situ hybridization. Specific staining is dark purple. Black arrows point to regions of transcript expression that is specific to regeneration; empty arrows indicate reduced level of expression. The original site of amputation is indicated by the black dotted line. Boxed region in C and I indicate area of histological section as shown in C′ and I′ respectively, accompanied by their respective schematics. All limbs are oriented proximal to the left and posterior uppermost, with the tadpoles lying on their right side. aec, apical epidermal cap; fa, functional aec; b, blastema.

Specific *sulf* transcript expression was also observed in regenerating hindlimbs that were originally amputated at the future knee level at stage 52. Transient regeneration‐specific patterns of expression of the two *sulfs* were consistently observed in the redeveloping AER as well as the proliferating blastema, albeit during different phases of regeneration (Fig. 3J′). *Sulf1* transcripts were detected in the distal mesenchyme close to the AER at 1 day post‐amputation (dpa) (Fig. 3A). The distal expression of the gene persisted at 2 dpa, followed by a decrease in the level of expression at 3 dpa and was no longer visible in the blastema region by 4 dpa (Fig. 3B−D). The overall expression pattern of *sulf1* in the 4−6 dpa sample represented the developmental pattern of expression in the corresponding limb stages during development (Fig. 2D−F).

In comparison, expression of *sulf2* transcript in the blastema region of the regenerating limbs only became visible later at 2 dpa (Fig. 3I). The level of expression in the blastema region increased at 3 dpa, and much more by 4 dpa as staining for the transcript appeared to expand proximally with an apparent emphasis on the anterior side (Fig. 3J−K). By 5−6 dpa, the transcript expression in the anterior half of the regenerating structure became much more prominent, while the level of posterior expression decreased considerably (Fig. 3L−M). The expression pattern of the gene in samples at later days post‐amputation recapitulate its pattern of expression during developmental stage 51−53 (Fig. 2E−F).

## Discussion

### Unique patterns of *Xenopus Sulf*s expression in developing limbs

The ability of heparan sulfatases to modulate interaction between HSPGs and their various ligands enables sulfs to regulate multiple developmental pathways, not only during embryogenesis but also in the organogenesis of limbs and other organ tissues during later stages of animal development. *Sulfs*, particularly *sulf1*, show distinct expression in the developing appendages of vertebrates. The initial expression of the mammalian and avian *sulf1*s are rather different in the early limb/wing buds (Zhao et al. 2006; Langsdorf et al. 2007; Ratzka et al. 2008; Otsuki et al. 2010). The expression of murine *sulf1* in E10.5 mice limb buds appeared to coincide with the expression of *fgf‐8* in the AER, whereas expression of the quail *sulf1* is seen primarily in the anterior−proximal part of the early stage 24−26 quail limb buds (Zhao et al. 2006; Ratzka et al. 2008). In subsequent developmental stages, the respective *sulf1*s are shown to be expressed in the condensing mesenchyme, cartilage, perichondrion and the developing limb joints in both groups of models (Zhao et al. 2006; Ratzka et al. 2008). The expression pattern of the amphibian *sulf1* in the developing joints of *X. laevis* limbs during later stages of development showed remarkable similarities to the aforementioned models. The expression of the gene in the earlier limb buds, however, differs significantly from the murine and avian models. We failed to detect any transcript expression in the AER region of the early limb buds, and while the expression of sulf1 does show an anterior bias later in development, the initial pattern of expression in the posterior end of stage 50−51 limb buds does not.

The overall pattern of expression of the murine *sulf1* and *sulf2* overlaps in the developing limb joints in younger limbs (Ratzka et al. 2008). The expression of mouse *sulf2* compared to mouse *sulf1*, however, demonstrated broader expression throughout the developing limbs, and was detected everywhere but the AER region of the E10.5 limb buds (Ratzka et al. 2008). The overlapping expression patterns overall and an additional functional study showing penetrance and severity of skeletal malformation phenotypes increasing with reduced number of alleles indicate redundant functions of the two mouse *sulf*s in the fine‐tuning of developmental structure in the appendages (Ratzka et al. 2008). In contrast, the expression patterns of the two *Xenopus sulf*s show marginal similarities past the initial limb bud stages (stage 51). While both genes demonstrated an emphasis in anterior expression, the expression of the *Xenopus sulf2* in the later limb stages was primarily seen in the interdigital space of the developing autopod, and was altogether absent from the joints. The expression of the two *Xenopus* sulfs therefore appeared to be more complementary than they were overlapping. The apparent differences between the domains of expression of the *Xenopus* and the murine *sulf*s suggest a unique function of the genes in the amphibian model that is far from redundancy. As the expression of *Xenopus sulf2* in forelimbs regressed significantly and was much more transient compared to its hindlimb expression, it is likely to be related to the development of the webbing structure in the amphibian. It will be interesting to see if the quail as well as chicken *sulf2* will demonstrate a similar expression pattern in the webbing of the respective autopods compared to *sulf2*.

### Differential *sulf* expression in regenerating hindlimbs

The murine *sulf*s were reported to be differently expressed in quiescent and activated SCs, which are an essential component of skeletal muscle regeneration in mammals (Langsdorf et al. 2007). The level of *sulf1* mRNA expression appeared to be higher in freshly isolated SCs, representing the initial onset of regeneration after injury. On the other hand, the expression of *sulf2* mRNA was absent in freshly isolated SCs and was only detected after 3 days of cell culture (Langsdorf et al. 2007). Subsequent functional studies using *sulf* double mutant mice demonstrated that mouse Sulfs have a redundant but important role in promoting muscle regeneration by controlling the proliferation‐to‐differentiation state of the SCs during regeneration through their influence over wnt signaling activities (Langsdorf et al. 2007; Tran et al. 2012; Maltseva et al. 2013). The apparent redundancy of the murine *sulf* function in regenerating tissues was also indicated in wound repairing in mice corneal epithelial cells (Maltseva et al. 2013).

The differential murine *sulf* expression shows remarkable similarity to the patterns of amphibian *sulf* expression in the blastema of the regenerating tadpoles’ hindlimbs observed in this study, as *Xenopus sulf1* transcripts in the blastema were only seen in the 1−3 dpa samples, whereas strong expression of *sulf2* was observed in 3−6 dpa samples. These consistencies suggest that, like the mouse *sulf*s, the *Xenopus sulf*s may play a role in promoting regeneration of the muscle tissues in *Xenopus* hindlimbs, probably by regulating the interaction of the canonical and non‐canonical Wnt signaling pathways that control the proliferating/differentiating state of the blastema (Tran et al. 2012).

However, considering that the developmental pattern of the *Xenopus sulf2* expression is different from the documented expression pattern of mouse *sulf2*, the two *Xenopus sulf*s may not be functionally redundant in the regenerating structure. Instead, the differential intensity as well as the timing of the two *sulf* expressions during regeneration may reflect their alternate roles in directing the proliferating or differentiating states of the blastema. In mice, the inductive function of Sulf1 over SC differentiation and muscle regeneration via the canonical Wnt signaling pathway is growth factor dependent, and the expression of Sulfs are known to inhibit Fgf signaling while enhancing Bmp in chondrocytes (Langsdorf et al. 2007; Otsuki et al. 2010). Expressions of *X. tropicalis sulf1* and *sulf2* are also known to restrict Bmp and Fgf signaling in developing embryos (Isaacs et al. 1992; Hongo et al. 1999; Freeman et al. 2008). As regeneration‐specific expression of *X. laevis sulf1* is only seen in the early phases of regeneration, in contrast to the expression of *sulf2* in the later part of the 6‐day regeneration period, it is likely that *sulf2* plays a more prominent role in modulating the Fgf as well as Bmp related activities during the redevelopment phase of limb regeneration. The amphibian *sulf1*, on the other hand, may be more involved in driving the initial proliferation and differentiation of the blastema cells, as suggested by the role of the mouse Sulf1 in regulating growth factor signaling for SC differentiation and the enhancement of myoblast fusion in injured tissue in mice (Langsdorf et al. 2007).

## Experimental Procedures

### RNA probe synthesis and in situ hybridization

Primers that cover 80%−100% of *X. laevis sulf1* and *sulf2* coding regions were designed based on the nucleotide details NM_001097379.1 and NM_001094945.1 respectively from the NCBI database. The 6‐*O*‐endosulfatase genes were amplified from cDNA reverse transcribed from RNA extracted from stage 38 and 42 *X. laevis* embryos. PCR products of the respective genes were cloned into pCRIITOPO TA vector (supplied by Invitrogen NZ) and the plasmids were linearized via PCR with the M13 sequencing primers prior to RNA probe synthesis. Digoxigenin labelled RNA probes were synthesized and whole mount in situ hybridization was carried out following procedures previously described by Pownall et al. (1996) with modification for limb samples as in McEwan et al. (2011).

Samples were photographed in phosphate buffer solution (PBS) on 1.5%−2% noble agar using a Leica Fluo III dissecting microscope and accompanying Leica camera and LS software. All figures were processed and compiled in Adobe Photoshop CS5.

### Histology

Fixed specimens of Sulf in situ hybridization were gradually dehydrated with various concentrations of ethanol prior to standard embedding procedures for paraffin wax. The paraffin blocks were subjected to serial sectioning at 8−10 μm using a Leica RM2125RT microtome. Permanent mounting of the slides was accomplished using water‐based Immu‐Mount and sealed with generic nail polish. Photographs of all histological sections were taken using a Zeiss Axiphot compound microscope with an attached Sony camera.

### Limb operation for regeneration experiment

All procedures were approved by the University of Otago animal ethics committee (AEC#56/12). Anesthetized tadpoles (1/4000 v/v MS222 in 0.1 × MMR) were laid down on their left side, with their right limbs exposed, on damp paper towels. The right hindlimb was removed at the future knee level using cannas iridectomy scissors. Operated tadpoles were allowed to revive in a recovery tank filled with 0.1 × MMR and aeration until normal swimming was observed. These were then returned to allocated tanks in a Marine Biotech XR3 aquarium.

For fixation, tadpoles were subjected to terminal anesthesia (1/400 MS222), and then fixed in 4% paraformaldehyde for either 2 h for the blastema samples or 4 h for the limb development samples. Fixed samples were dehydrated in serial concentrations of ethanol in PBS, and stored in absolute ethanol at −30 ˚C until required.

## Supporting information

Additional Supporting Information may be found in the online version of this article at the publisher's website:

**Figure S1**. *In situ* hybridization of stage 35−40 *X. laevis* embryos to *Sulf1* and *Sulf2* anti‐sense probes. Specific staining is dark purple. ba, branchial arches; pn, pronephros; fp, floor plates; cg, cement gland; f, fin.
