# Supplementary figures and images for: Distinct patterns of endosulfatase gene expression during Xenopus laevis limb development and regeneration
Source: Regeneration (Oxf). 2015 Mar 13;2(1):19–25. doi: 10.1002/reg2.27 (PMC4895329; doi:10.1002/reg2.27)

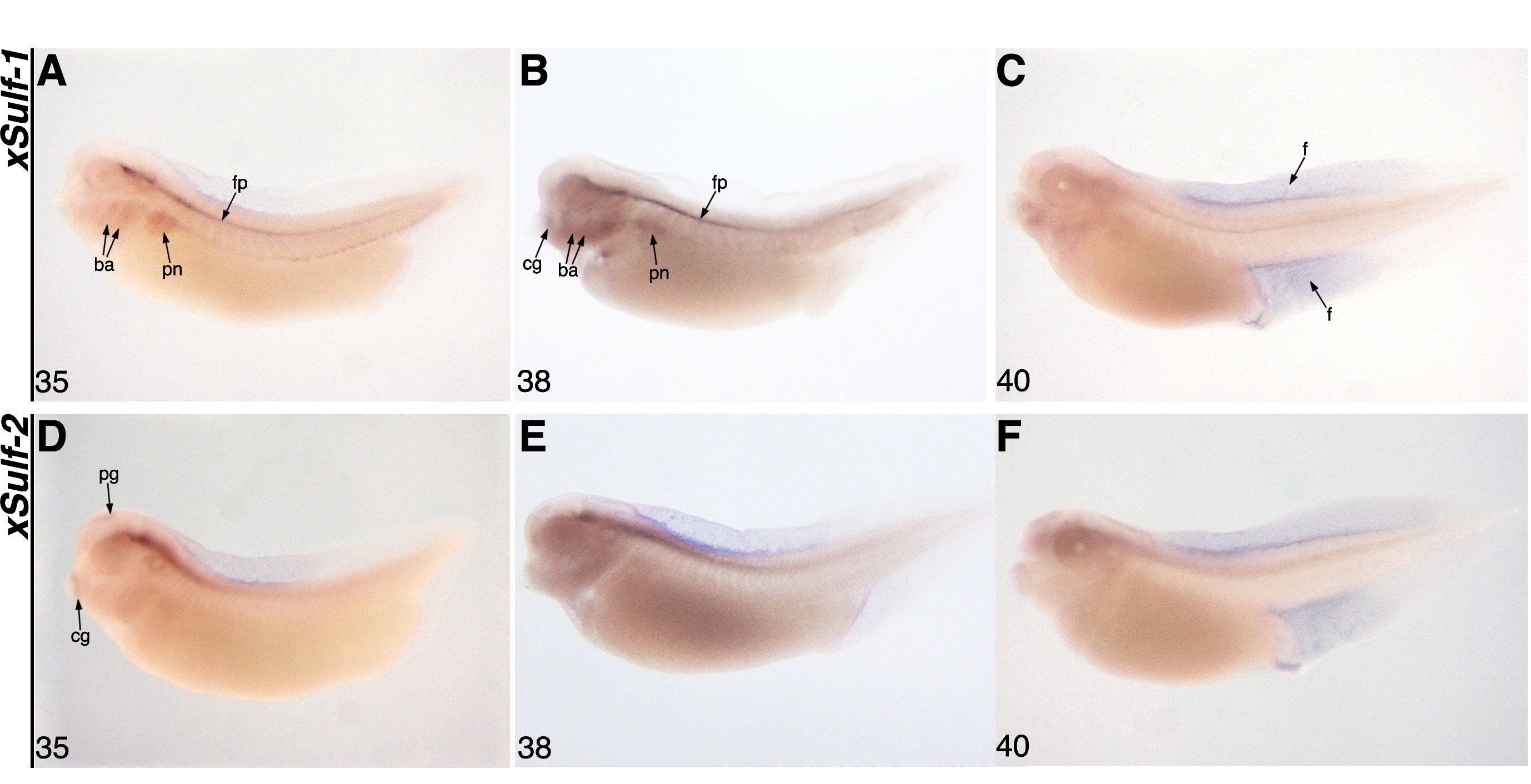

Supplement: Supplementary file 1 — Figure S1. In situ hybridization of stage 35−40 X. laevis embryos to Sulf1 and Sulf2 anti‐sense probes. Specific staining is dark purple. ba, branchial arches; pn, pronephros; fp, floor plates; cg, cement gland; f, fin. [file REG2-2-19-s001.tif]
